# Phosphorylation of CBP20 Links MicroRNA to Root Growth in the Ethylene Response

**DOI:** 10.1371/journal.pgen.1006437

**Published:** 2016-11-21

**Authors:** Fan Zhang, Likai wang, Jae Yun Lim, Taewook Kim, Youngjae Pyo, Sibum Sung, Chanseok Shin, Hong Qiao

**Affiliations:** 1 Institute for Cellular and Molecular Biology, The University of Texas at Austin, Austin, Texas, United States of America; 2 Department of Molecular Biosciences, The University of Texas at Austin, Austin, Texas, United States of America; 3 Department of Agricultural Biotechnology, Seoul National University, Gwanak-gu, Seoul, Republic of Korea; 4 Research Institute of Agriculture and Life Sciences, and Plant Genomics and Breeding Institute, Seoul National University, Seoul, Republic of Korea; Peking University, CHINA

## Abstract

Ethylene is one of the most important hormones for plant developmental processes and stress responses. However, the phosphorylation regulation in the ethylene signaling pathway is largely unknown. Here we report the phosphorylation of cap binding protein 20 (CBP20) at Ser^245^ is regulated by ethylene, and the phosphorylation is involved in root growth. The constitutive phosphorylation mimic form of *CBP20* (*CBP20*^*S245E*^ or *CBP20*^*S245D*^), while not the constitutive de-phosphorylation form of *CBP20* (*CBP20*^*S245A*^) is able to rescue the root ethylene responsive phenotype of *cbp20*. By genome wide study with ethylene regulated gene expression and microRNA (miRNA) expression in the roots and shoots of both Col-0 and *cbp20*, we found miR319b is up regulated in roots while not in shoots, and its target *MYB33* is specifically down regulated in roots with ethylene treatment. We described both the phenotypic and molecular consequences of transgenic over-expression of *miR319b*. Increased levels of *miR319b* (*miR319bOE*) leads to enhanced ethylene responsive root phenotype and reduction of *MYB33* transcription level in roots; over expression of *MYB33*, which carrying mutated miR319b target site (*mMYB33*) in *miR319bOE* is able to recover both the root phenotype and the expression level of *MYB33*. Taken together, we proposed that ethylene regulated phosphorylation of CBP20 is involved in the root growth and one pathway is through the regulation of miR319b and its target *MYB33* in roots.

## Author Summary

Ethylene is one of the most essential hormones for plant developmental processes and stress responses. However, the phosphorylation regulation in the ethylene signaling pathway is largely unknown. Here we found that ethylene induces the phosphorylation of CBP20 at S^245^, and the phosphorylation is involved in root growth. Genome wide study on ethylene regulated gene expression and microRNA expression together with genetic validation suggest that ethylene- induced phosphorylation of CBP20 is involved in root growth and one pathway is through the regulation of miR319b and its target gene *MYB33*. This study provides evidence showing a new link of cap binding protein phosphorylation associated microRNA to root growth in the ethylene response.

## Introduction

The plant hormone ethylene (C_2_H_4_) is essential for a myriad of physiological and developmental processes [1–3]. A linear ethylene signaling pathway has been established [4] that plants perceive ethylene by ER-located receptors, which are similar to the bacterial two component histidine kinases [5, 6]. In the absence of ethylene, the receptors activate a Raf-like protein kinase CONSTITUTIVE TRIPLE RESPONSE 1 (CTR1) [7]. Activated CTR1 inhibit an ER-tethered protein ETHYLENE INSENSITIVE 2 (EIN2) through phosphorylation [8, 9]. EIN2 is degraded and the degradation is mediated by two F-box proteins: ETP1 and ETP2 [10]. In the presence of ethylene, the EIN2 C-terminal (EIN2-C) is dephosphorylated, cleaved and translocated into both the nucleus and P-body [9, 11, 12]. In the nucleus, the EIN2 CEND transduces signals to the transcription factors ETHYLENE INSENSTIVE3 (EIN3) and ETHYLENE INSENSITIVE3-LIKE1 (EIL1), which are sufficient and necessary for activation of all ethylene-response genes [13, 14]. In P-body, EIN2C mediates translation repression of EBF1 and EBF2 [15, 16], which are the two F-box proteins, which target EIN3 for degradation [17, 18]. Recently new study discovered that noncanonical histone acetylation H3K23Ac is involved in ethylene regulated gene activation in an EIN2 and partial EIN3 dependent manner [19].

Protein phosphorylation plays critical roles in ethylene response. Such as ethylene receptors are similar in sequence and structure to bacterial two-component histidine kinases, and ethylene controls autophosphorylation of the histidine kinase domain in ethylene receptor ETR1 [20], the histidine kinase activity of ETR1 is not required for but plays a modulating role in the regulation of ethylene responses [21]. Furthermore, biochemical and functional analysis of CTR1, a protein similar to the Raf family protein kinases that negatively regulates ethylene signaling in *Arabidopsis* [22]. Recently study has demonstrated that in the absence of ethylene, CTR1 targets to EIN2 C-terminal end for phosphorylation with its kinase domain [9]. However, the phosphorylation regulation in ethylene signaling is still under developed.

In this study, we found that ethylene induces phosphorylation of CBP20 at Ser^245^. Constitutive phosphorylation mimic form of CBP20^S245D^ or CBP20^S245E^ rescues the root less sensitive phenotype of *cbp20* mutant in ethylene, but the constitutive dephosphorylation mimic form of CBP20^S245A^ is unable to rescue *cbp20* mutant phenotype. Through small RNA sequencing and mRNA sequencing, we found a set of miRNAs and their targets are specifically regulated in roots by ethylene in a CBP20 dependent manner. Among them, the expressions of *miR319b* and its potential target *MYB33* display anti-correlation pattern in a CBP20 dependent manner in Col-0 roots. Small RNA northern blot in roots shows that miR319 is specific up regulated by ethylene treatment, which requires CBP20 phosphorylation. Genetic study shows that over expression of *miR319b* leads to the down regulation of *MYB33*, resulting in enhanced ethylene sensitive phenotype in roots, which is similar to its target *myb33* mutant phenotype. The phenotype of *miR319bOE* is rescued by adding mutated *MYB33 (mMYB33)*, which containing mutation at miR319 targeting site. Furthermore, we provided evidence showing that miR319b, while not miR159 influences the expression of *MYB33* in the presence of ethylene in roots. Overall, our results demonstrate that ethylene regulated phosphorylation of CBP20 is involved in the root growth. One model is through the regulation of miR319b and its target *MYB33* in roots in response to ethylene, providing a new link of cap binding protein phosphorylation associated microRNA to root growth in the ethylene response.

## Results

### Ethylene regulated phosphorylation of CBP20 is involved in root growth in ethylene response

**Fig 1:**
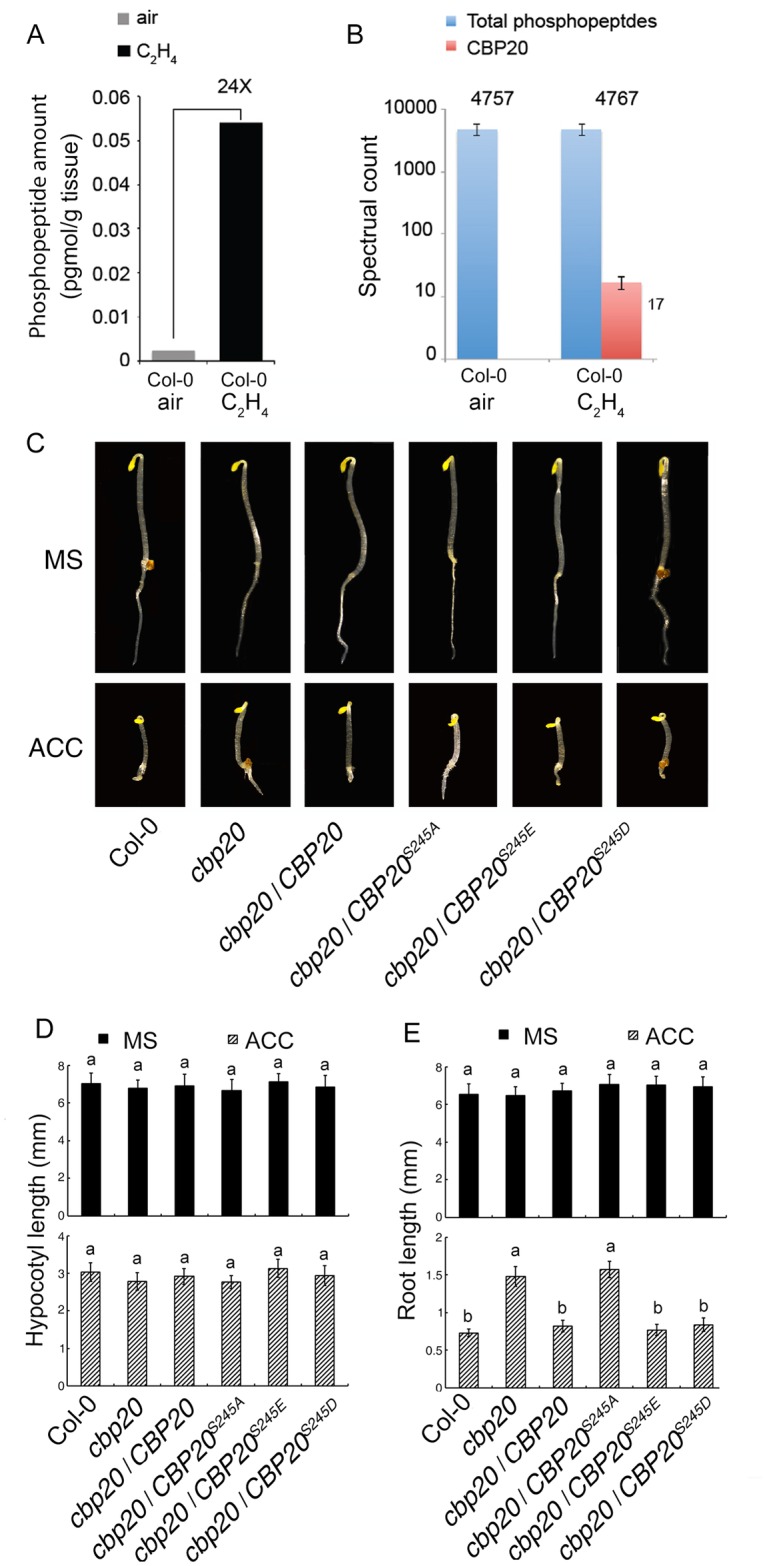
CBP20 phosphorylation is required for root development in ethylene response. (A) Absolute amounts of total phosphopeptides before and after treatment with 10 ppm ethylene gas. (B) Relative phosphorylation levels of CBP20 peptides in Col-0 or *ctr1-1* plants treated for 4 hours of air or 10 ppm ethylene gas. Spectral counts were computed by averaging three biological replicates. The total spectral counts from all phosphorylated proteins in each sample are indicated as an internal control. (C) Triple response phenotype of *cbp20* mutant and *cbp20* mutant transformed with full length of CBP20, constitutive dephosphorylated of CBP20 (CBP20^S245A^) and constitutive phosphorylated of CBP20 (CBP20^S245E^ and CBP20^S245D^). (D-E) The length of hypocotyls (D) and roots (E) of *cbp20* mutant and *cbp20* mutant transformed with full length of CBP20, constitutive dephosphorylated of CBP20 (CBP20^S245A^) and constitutive phosphorylated of CBP20 (CBP20^S245E^ and CBP20^S245D^) on 10μm ACC plate for 3 days. Bars indicates SD and n = 30 for each line. The same letters indicates no statistic significant difference; the different letters indicate statistic significant difference with p≤ 0.05.

Previous studies have shown that phosphorylation plays critical roles in ethylene signaling and many ethylene regulated phosphorylation proteins have been identified [11, 23]. By searching the phosphorylation MS/LS data under ethylene treatment, we found that the CBP20, a component of cap-binding complex, is highly phosphorylated at Ser^245^ site with ethylene treatment (Fig 1A and 1B and S1A Fig). Protein alignment with CBP20s from different species showed that CBP20 is highly conserved, and the Ser245s are all identical through different species examined (S1B Fig), suggesting the function of CBP20 is conserved and the phosphorylation at S^245^ of CBP20 is potentially important for CBP20.

To study the role of phosphorylation of CBP20 in the regulation of ethylene response, the ethylene response phenotype of *cbp20* mutant was examined. We found *cbp20* mutant displayed partial reduced ethylene responsive phenotype in the roots, but not in the hypocotyls and apical hooks (Fig 1C–1E). To examine the connection between phosphorylation state of CBP20 and the ethylene responsive phenotype of *cbp20*, 35S promoter driven phosphorylation mimic form of *CBP20* (*35S*:*CBP20*^*S245D*^ and *35S*:*CBP20*^*S245E*^*)* and dephosphorylation mimic form of *CBP20* (*35S*:*CBP20*^*S245A*^) (S2A Fig), were generated and introduced into *cbp20* mutant to obtain *CBP20*^*S245A*^*/cbp20*, *CBP20*^*S245D*^*/cbp20* and *CBP20*^*S245E*^*/cbp20*. Additionally, 35S promoter driven wild type *CBP20* (*35S*:*CBP20*) was introduced to *cbp20* as control (S2A Fig). The full length of *CBP20*, *CBP20*^*S245E*^ and *CBP20*^*S245D*^ were able to rescue the *cbp20* mutant phenotype in the presence of ethylene. However, *CBP20*^*S245A*^ was unable to rescue the phenotype (Fig 1C–1E), which suggests that the phosphorylation of CBP20 is involved in the root growth in the presence of ethylene.

To explore how the phosphorylation of CBP20 is involved in the regulation in ethylene response, we first examined the root phenotype of *cbp80* mutant in response to ethylene. We found that *cbp80* displays similar phenotype as *cbp20* in the presence of ethylene (S2B Fig). We next tested the interaction between wild type CBP80 with CBP20, *CBP20*^*S245A*^, *CBP20*^*S245D*^ or *CBP20*^*S245E*^ by yeast two-hybrid. In consistent with previous study [24], we were able to detect the interaction between CBP20 and CBP80, however, the interaction was not influenced by the phosphorylation states of CBP20 (S2C Fig). Generally, CBP80 interacts with CBP20 and in assisting CBP20 transfer into nucleus [25], we then examined cellular localization of *CBP20*^*S245A*^*-YFP and CBP20*^*S245D*^*-YFP or CBP20*^*S245E*^*-YFP* into *cbp20* with or without the presence of ethylene. Both wild type CBP20 and mutated CBP20 were mainly localized in the nucleus and their localizations were not altered by ethylene treatment (S2D Fig).

### Small RNA and RNA sequencing analysis of CBP20 regulated microRNAs in roots with ethylene treatment

**Fig 2:**
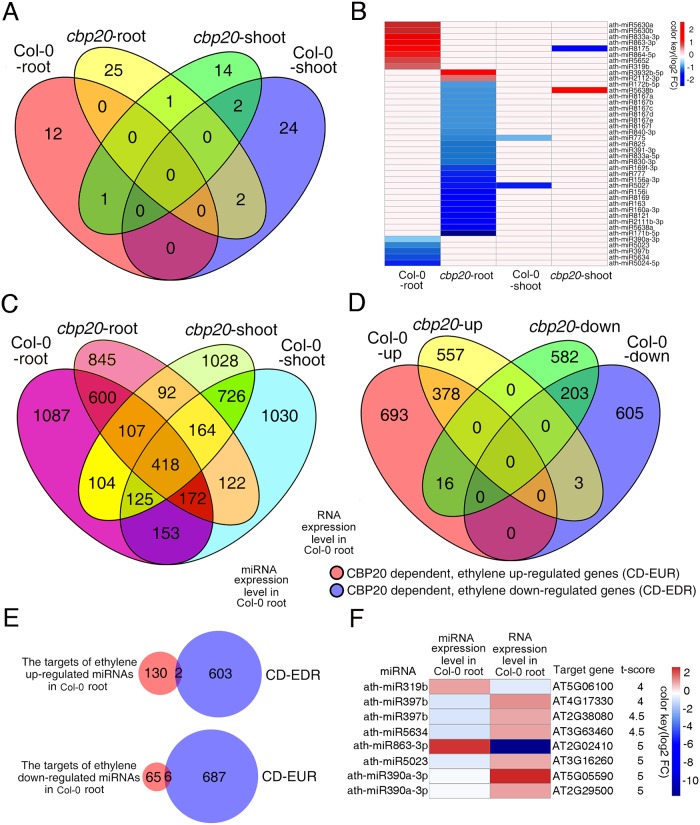
Small RNA and RNA sequencing analysis of CBP20 regulated miRNAs and RNAs in roots and shoots with ethylene treatment. (A) The Venn diagram shows the numbers of ethylene regulated miRNAs in the roots or shoots of Col-0 and *cbp20* mutant. The miRNAs, which are differentially regulated by ethylene in the shoots or roots of Col-0 or *cbp20* are compared. (B) Heat map shows the expression level of differential regulated miRNAs in the root and shoot of Col-0 and *cbp20* mutant with ethylene treatment. Total small RNA was prepared from the roots or shoots of 3-day-old etiolated seedlings of Col-0 or *cbp20* plants treated with air or 4 h ethylene. Differentially expressed miRNA requiring a 1.5 fold change comparing the indicated conditions with P<0.05 after Benjamini–Hochberg correction. (C) The Venn diagram shows the numbers of ethylene regulated genes in the roots and shoots of Col-0 and *cbp20* mutant. Total RNA was prepared from the roots or shoots of 3-day-old etiolated seedlings of Col-0 or *cbp20* plants treated with air or 4 h ethylene. Differentially expressed genes were identified by fragments per kilobase per million reads (FPKM) filter<0.1, requiring a twofold change comparing the indicated conditions with P<0.05 after Benjamini–Hochberg correction (D) The Venn diagram shows the number of The CBP20 dependent differential regulated genes in Col-0 root after ethylene treatment. (E) The venn diagram shows the number of the targets of differential regulated miRNAs and the CBP20 dependent differential regulated genes in Col-0 roots after ethylene treatment. (F) Heat map shows the expression level of differential regulated miRNAs and their target genes in col root after ethylene treatment.

CBC complex has a key role in several gene expression mechanisms [26–28] and CBP20 is essential for miRNA biogenesis [29–31]. We speculated that CBP20 is required for the biogenesis of miRNAs in the root growth in ethylene response. To address this question, we conducted small RNA sequencing using the roots and shoots isolated from 3-day old etiolated seedlings of Col-0 or *cbp20* mutant treated with air or ethylene (S3A and S3B Fig). In consistent with previous study [30], most of species of miRNA detected were down regulated in *cbp20* (S1–S4 Tables). By comparing the miRNA expressions in the roots and shoots of Col-0 and *cbp20* treated with air or 4 hours ethylene gas. We found that ethylene altered miRNA expressions in a tissue specific manner (Fig 2A). As shown in Fig 2A and 2B, almost no shared ethylene induced differential expressed miRNAs were detected in the shoots and roots in Col-0 or *cbp20* mutant. Given ethylene regulated miRNA expression is tissue specific, and *cbp20* root specific phenotype with ethylene treatment, we speculated that the ethylene responsive root phenotype of *cbp20* potentially due to the alteration of miRNAs in roots. Through further analysis, 13 ethylene regulated miRNAs (P<0.05) were identified specifically in Col-0 roots (Fig 2A). Among them, 7 miRNAs were up regulated and 5 were down regulated, and most of them are uncharacterized miRNAs (Fig 2B and S1 Table). Overall these results demonstrate that ethylene alters miRNA expression in a tissue specific manner, and we are able to identify ethylene regulated miRNAs specifically in roots in a CBP20 depend manner.

In plants, the main function of miRNAs appears to be in gene regulation. Therefore, we expected the expression of the potential targets of 12 miRNAs identified above is anti-correlated with their miRNAs in response to ethylene in Col-0 roots. We conducted RNA sequencing use the same tissues as mentioned in small RNA seq with two biology duplications (S4A and S4B Fig). Comparable numbers of ethylene-regulated genes were detected in the roots and shoots of Col-0; however, only about 20–30% of genes were overlapped between these two type tissues (Fig 2C and S1–S4 Datasets), showing the tissue specific in ethylene response. Further GO analysis showed that ethylene related GO terms were enriched in those ethylene regulated genes shared between shoots and roots, and root development related GO terms were enriched in the genes specifically regulated in roots (S5A–S5C Fig). Similarly, in *cbp20* mutant the gene regulation also showed tissue specificity in response to ethylene (Fig 2C and S1–S4 Datasets).

We then compared ethylene regulated gene expression in the roots of Col-0 and *cbp20*. As shown in Fig 2D, about 60% of up regulated genes and 75% of down regulated genes in Col-0 roots were not altered in *cbp20* roots in the presence of ethylene (S5 and S6 Datasets), showing CBP20 dependency. We then studied the association between 12 microRNAs and their targets genes in ethylene response. In total 841 potential target genes were identified and 203 with high confidence (T score < = 5) (S7 Fig and S8 Dataset). Among them, only 8 target genes were differentially regulated by ethylene in the roots of Col-0, while their differential expressions were impaired in *cbp20* (Fig 2E). By comparing the expressions of ethylene altered miRNAs and their target genes in Col-0 roots, two miRNAs (miR319b, miR863-3p) were identified that up regulated by ethylene and the expression of their potential target genes was down regulated in the presence of ethylene in the roots of Col-0, while not in the roots of *cbp20* (Fig 2F).

**Fig 3:**
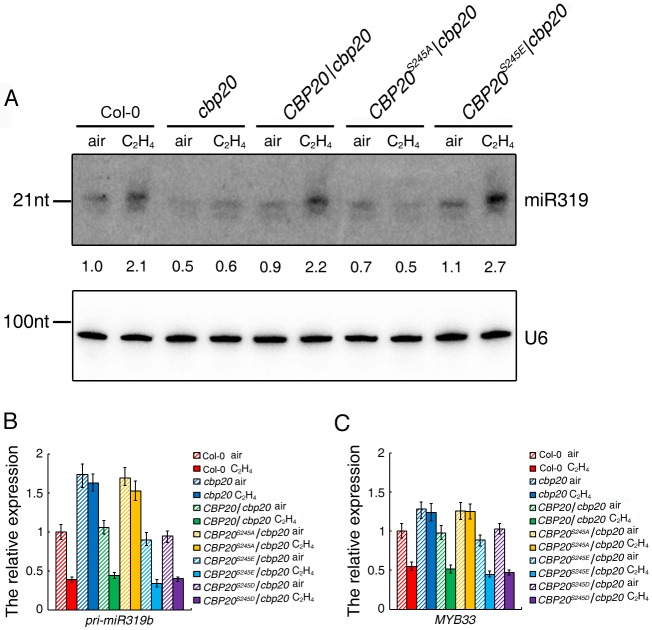
CBP20 phosphorylation regulates miR319 biogenesis and *MYB33* expression. (A) Small RNA northern blot of miR319 in roots of 3-day old seedlings of plants indicated in the figure treated with air or 4 hours ethylene gas. Numbers indicate the relative expression level compared with the U6 control. (B) The relative gene expression of *pri-miR319b* in roots of 3-day old seedlings of plants indicated in the figure treated with air or 4 hours ethylene gas. (C) The relative gene expression level of *MYB33* in *cbp20* mutant and cbp20 mutant transformed with full length of CBP20, constitutive mimic dephosphorylated of CBP20 (CBP20^S245A^) and constitutive mimic phosphorylated of CBP20 (CBP20S^245E^ and CBP20^S245D^) treated with ethylene. Total RNAs were extracted from the roots of 3-day-old etiolated seedlings from indicated genotypes and gene expression was analyzed by qualitative RT-PCR (3 biological replicates).

### Phosphorylation of CBP20 is required for the elevation of miR319 and down-regulation of *MYB33* expression in response to ethylene

To validate the function of miRNAs identified above in response to ethylene, and the connection between their expressions with the phosphorylation state of CBP20, we examined mature miR319 by northern blot in *cbp20* mutant, *CBP20*/*cbp20*, *CBP20*^*S245A*^*/cbp20* and *CBP20*^*S245E*^*/cbp20* with or without ethylene treatment. In consistent with small RNA sequencing result, miR319 indeed was up regulated by ethylene in the roots of Col-0 (Fig 3A), and the elevation was impaired in the roots of *cbp20* mutant. Furthermore, constitutive phosphorylated *CBP20*^*S245E*^, while not dephosphorylated *CBP20*^*S245A*^ were able to recover the ethylene induced elevation of miR319 expression (Fig 3A), indicating that ethylene induced phosphorylation of CBP20 potentially required for the elevation of miR319b expression in roots. We next examined the expression of *pri-miR319b* in the roots of Col-0, *cbp20*, *CBP20*/*cbp20*, *CBP20*^*S245A*^/*cbp20*, *CBP20*^*S245D*^/*cbp20* and *CBP20*^*S245E*^/*cbp20* treated with air or 4 hours ethylene gas by quantitative RT-PCR. As shown in Fig 3B, the expression of *pri-miR319b* was decreased with the ethylene treatment and the down regulation was impaired in *cbp20* mutant. The down regulation of *pri-miR319b* was detected in the roots of *CBP20*^*S245D*^*/cbp20* or *CBP20*^*S245E*^*/cbp20*, while not in that of *CBP20*^*S245A*^*/cbp20* (Fig 3B), indicating that the phosphorylation is required for the down regulation of pri-miRNA, further suggesting that the elevation of miR319b in response to ethylene due to the biogenesis of miRNA, while not due to the elevation of pri-miR319b.

To further examine how the phosphorylation of CBP20 influences the gene expression of *MYB33* in response to ethylene, we conducted qRT-PCR in the roots of Col-0, *CBP20*/*cbp20*, *CBP20*^*S245A*^*/cbp20*, *CBP20*^*S245D*^*/cbp20* and *CBP20*^*S245E*^*/cbp20* with or without ethylene treatment. The expression level of *MYB33* was indeed decreased by ethylene treatment, which is consistent with RNA-seq result (Fig 3C). In *cbp20* and *CBP20*^*S245A*^*/cbp20* plants, the down regulation of *MYB33* was impaired, however, in *CBP20*^*S245D*^*/cbp20* or *CBP20*^*S245E*^*/cbp20* (Fig 3C), the expression of *MYB33* is recovered as that of in Col-0. Overall, the result shows that the expression of *MYB33* is anti-correlated with the expression of miR319b specifically in roots in ethylene response, indicating that ethylene induced phosphorylation of CBP20 inhibits the expression of *MYB33*, which potentially through CBP20 regulated biogenesis of miR319b in roots.

### *myb33* mutants and overexpression of *miR319b* plants display the ethylene hypersensitive phenotype in roots

**Fig 4:**
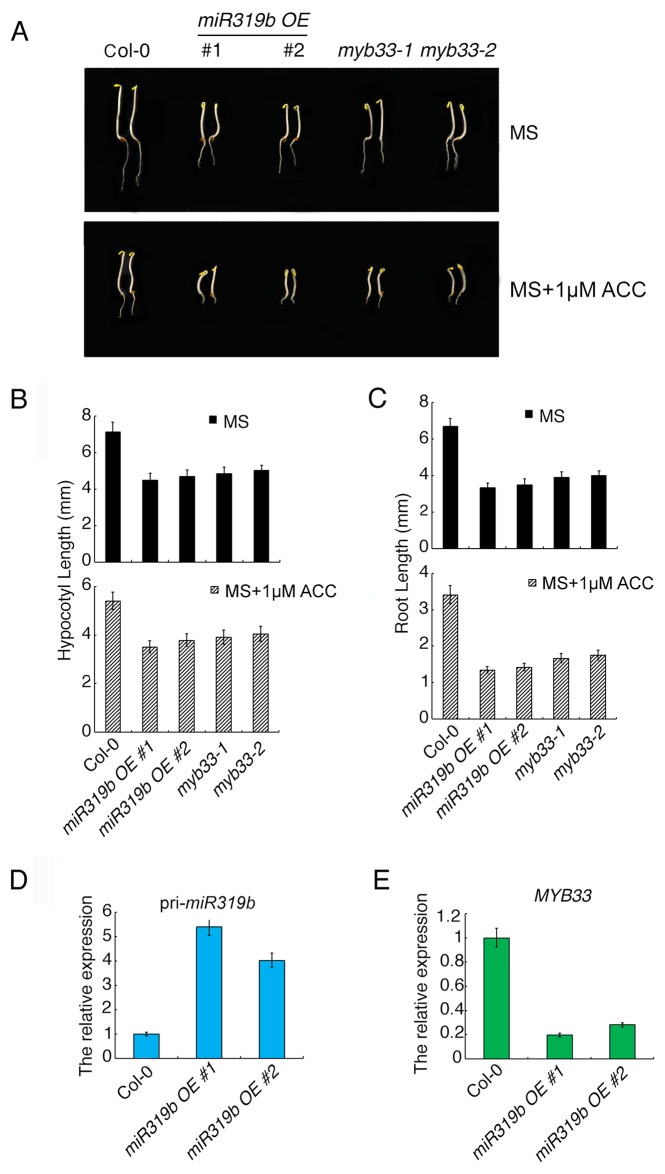
*miR319bOE* plants and *myb33* mutants show the ethylene hypersensitive phenotype. (A) Triple response phenotype of *miR319b OE* plants and *myb33* mutants. (B-C) The length of hypocotyls (B) and roots (C) of *miR319b OE* plants and *myb33* mutants grown on MS or MS with 1μM ACC. (D-E) The relative expression level of *pri-miR319b* (D) and *MYB33* (E) in *miR319bOE* plants. Total RNAs were extracted from the roots of 3-day-old etiolated seedlings from indicated genotypes and gene expression was analyzed by qRT-PCR (3 biological replicates).

To further examine whether miR319b plays a role in root growth in ethylene response, we generated the *miR319b* overexpression (*miR319bOE)* plants, and examined their phenotype in response to ethylene. As expected, the roots of *miR319bOE* plants were more sensitive to ethylene than that of wild type (Fig 4A–4C). *MYB33* is one of potential targets of miR319b, we therefore obtained *myb33* mutant to examine its phenotype in response to ethylene. As expected, the roots of *myb33* mutant displayed similar phenotype as that of *miR319bOE* in the presence of ethylene (Fig 4A–4C and S6A and S6B Fig). Comparing to wild type, the pri-miR319b was increased (Fig 4D), while the expression of *MYB33* was decreased in *miR319bOE* plants (Fig 4E), showing that the elevation of miR319b is not due to the elevation of its precursor in the presence of ethylene, but potentially due to the miRNA biogenesis process in response to ethylene. We then conducted a 5′ RNA Ligase-Mediated (RLM)-Rapid Amplification of cDNA ends (RACE) in both Col-0 and *miR319OE* plants to evaluate that *MYB33* is one of targets of mir319b *in vivo*, In Col-0, no cleavage event was detected between the 10th nucleotide U and the 11nd nucleotide C from the 5′ end of the miRNA in Col-0. However, in *miR31bOE*, 5 out of 15 cleavage events were detected between nucleotides 10 and 11 from the 5′ end of the miRNA (S6C Fig), which are in consistent with the published data [32]. Taken all together, these results indicate that miR319b is involved in root growth by targeting *MYB33* for degradation in a CBP20 dependent manner.

**Fig 5:**
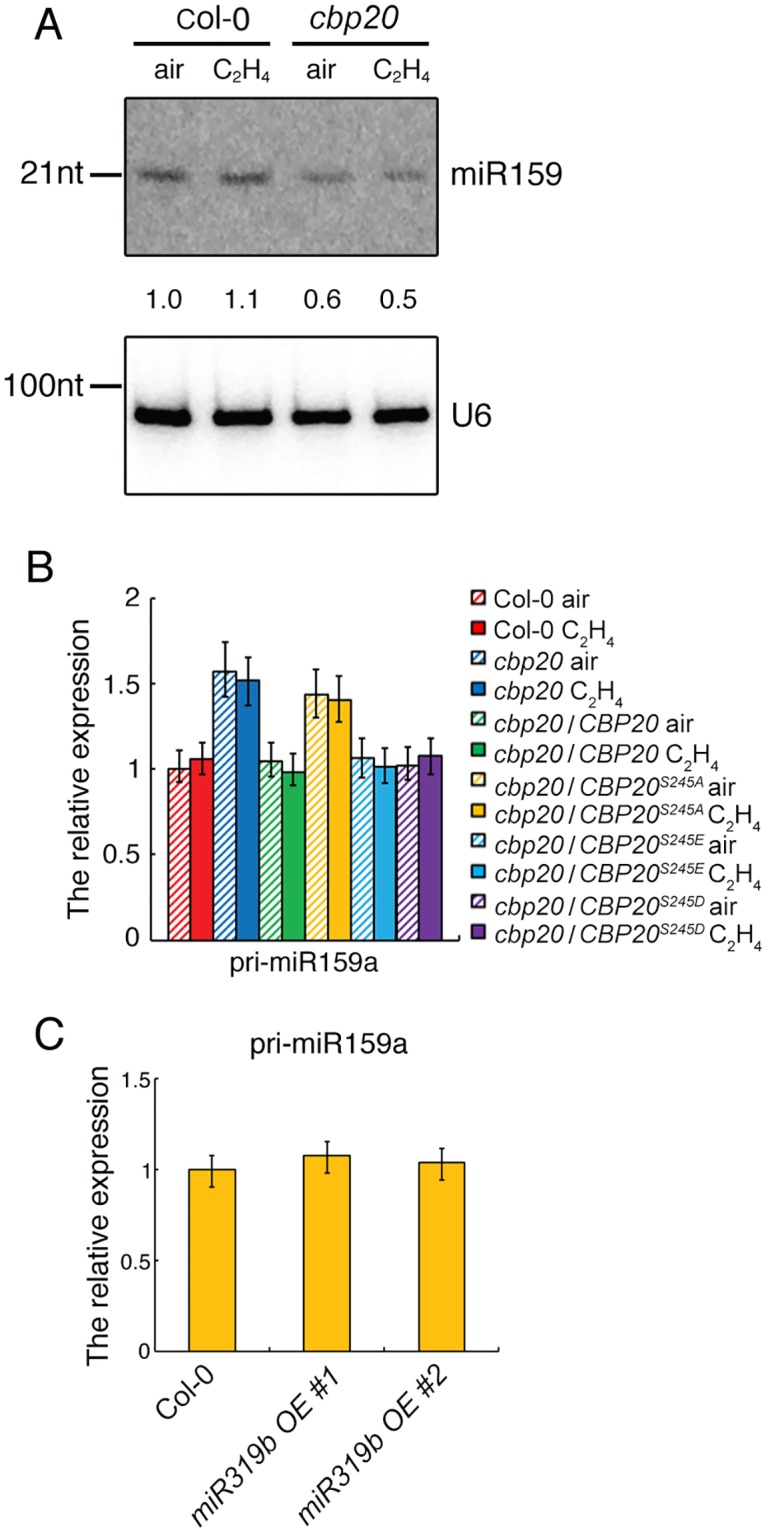
CBP20 phosphorylation does not influence expression of miR159. (A) Small RNA northern blot of miR159 in Col-0 and *cbp20* mutant treated with or without 4 hours ethylene gas. Numbers indicate the relative expression level of miR159 compared with the U6 control. (B) The relative gene expression level of *pri-miR159a* in *cbp20* mutant and *cbp20* mutant transformed with full length of *CBP20 (CBP20/cbp20)*, constitutive dephosphorylated of *CBP20* (*CBP20*^*S245A*^*/cbp20*) and constitutive phosphorylated of CBP20 (*CBP20*^*S245E*^*cbp20* and *CBP20*^*S245D*^*/cbp20*) treated with or without 4 hours ethylene gas. (C) The relative expression level of *pri-miR159a* in *miR319bOE* lines.

Because *MYB33* is a shared target between miR319 and miR159, we examined the expression of miR159 in the roots of Col-0 and *cbp20* treated with air and ethylene by northern blot. Inconsistent with small RNA-seq result, no differential expression of miR159 was detected in Col-0 roots between air and ethylene treatments (Fig 5A). As previous published data [30], we detected the reduction of miR159 in *cbp20* comparing to that of in Col-0, which is consistent with published data [30]. However, no ethylene induced alteration for miR159 was detected in the roots of both Col-0 or *cbp20* mutant (Fig 5A). In addition, no significant difference of pri-miR159 was detected in both Col-0 and *cbp20* roots by ethylene treatment as well (Fig 5B). Furthermore, the phosphorylation mimic forms of CBP20^S245D^ and CBP20^S245E^, while not dephosphorylation mimic form of CBP20^S245A^ behaved as wild type CBP20 (Fig 5B). We further examined the *pri-miR159a* in two independent *miR319bOE* plants and found *pri-miR159a* was not affected by the overexpression of *miR319b* (Fig 5C), indicating that in the presence of ethylene, the down regulation of *MYB33* is associated with the up regulation of miR319b, while not miR159.

**Fig 6:**
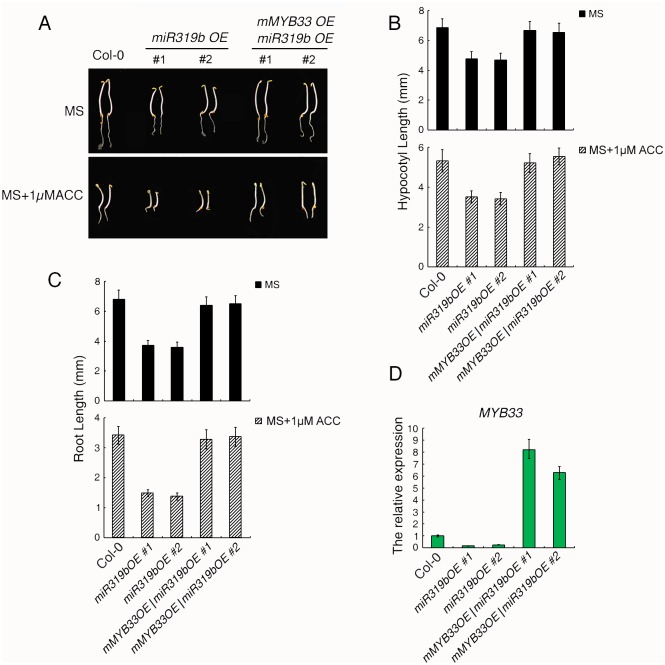
Mutated *MYB33* with miR319b target sites is able to rescue the root phenotype caused by *miR319bOE*. (A) Triple response phenotype of miR319bOE plants, *miR319bOE/mMYB33OE* plants. (B-C) Measurement of hypocotyls (B) and roots (C) of *miR319bOE* and *miR319bOE/mMYB33OE* plants. (D) The relative gene expression of *MYB33* in *miR319bOE* and *miR319bOE/mMYB33OE* plants. Total RNAs were extracted from the roots of 3-day-old etiolated seedlings from indicated genotypes and gene expression was analyzed by qualitative RT-PCR. Three replications have been done with similar result.

**Fig 7:**
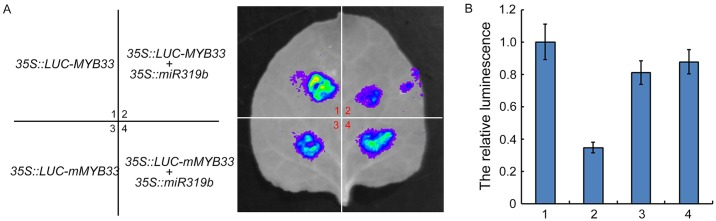
*MYB33* is miR319b target. (A) Luciferase assays showing that gene expression of *MYB33* is down regulated in the presence of miR319b precursor. *Agrobacterium* containing *MYB33* or mutated m*MYB33* CDS fused to 35S::LUC 3’UTR with or without *Agrobacterium* harbouring constructs containing the *miR319b* precursor were injected in to *N*. *benthamiana* plants. After 3 days, The leaves were sprayed with 500 μM luciferin and placed in the dark for 5 min. Luciferase activity was observed. (B) Quantitative measurement of luciferase intensity in different treatments as indicated in the figure. 3 biology replications have been done.

To evaluate whether the phenotype of *miR319bOE* is caused by down regulation of *MYB33*, we constructed mutated *MYB33* (*mMYB33*) carrying mutation in miR319 targeting site (S7A Fig) and introduced it into *miR319bOE* plants to obtain *miR319bOE/mMYB33OE*. The ethylene responsive phenotypes of both the roots and shoots of *miR31bOE* were recovered in *miR319bOE/mMYB33OE* plants (Fig 6A–6C). We then examined the expression of *MYB33* in the roots of *miR319bOE* and *miR319bOE/mMYB33OE*. In consistent with Fig 5E, the expression of *MYB33* was down regulated in *miR319bOE* plants, and was highly up regulated in *miR319bOE/mMYB33OE* in comparing to that of in Col-0 (Fig 6D), while the expression of *pri-miR319b* in *miR319bOE* and in *miR319bOE/mMYB33OE* plants were comparable (S7B Fig). TCPs are known targets of miR319, however, no ethylene induced alteration of TCPs was detected in our RNA-seq result, to further confirm the result, we examined the gene expression of *TCPs* by qRT-PCR in Col-0 treated with or without ethylene. No significant change was detected for those gene expressions in response to ethylene (S7C Fig). In addition, gene expression of *TCP24* was not altered in *miR319bOE/mMYB33* in comparing to that of in *miR319bOE* plants (S7D Fig), and the expression of other TCPs displayed similar patterns as *TCP24* both in Col-0 and *miR319OE* plants (S7E Fig). Finally we conducted Agrobacterium-mediated transient co-expression assay with *MYB33* or mutated m*MYB33* CDS fused to 35S::LUC 3’UTR with or without the *miR319b* precursor. Comparing to the assay without miR319b precursor, *MYB33* expression was significantly lower in the presence of miR319b precursor (Fig 7A and 7B). However, no significant change was detected for *mMYB33* expression between with and without the presence of miR319b precursor (Fig 7A and 7B). The similar assay was also conducted using YFP-HA-tagged *MYB33* or mutated *MYB33* (*mMYB33*) with or without miR319b precursor to examine how miR319b influence gene expression of *MYB33* and its protein level. The gene expression of *MYB33*, while not *mMYB33*, is down regulated in the presence of miR319b (S8A Fig). In consistent with gene expression, MYB33 protein is also lower in the assay with the presence of miR319b. However, mMYB33 protein was not altered by miR319b (S8B Fig). Taken all together our data support that miR319b targets to *MYB33* for degradation in roots.

## Discussion

### CBP20 phosphorylation regulates root growth in ethylene signaling

It has been well known that protein phosphorylation are involved in many different plant hormones such as phosphorylation regulates the polarity of PIN in auxin [33–37], gibberellins [33, 38, 39], cytokinin [40], ABA [41] and in BR signaling [42–44]. Many studies have demonstrated that MPKKK cascade promotes ACS6 and EIN3 phosphorylation [45, 46]. Recently study has demonstrated that in the absence of ethylene, the receptors activate CTR1, which phosphorylates EIN2 C-terminus [9]. With the presence of ethylene, the EIN2C is dephosphorylated and then cleaved and translocated into nucleus to activate the downstream signaling pathway [11, 12]. However, the phosphorylation regulation in ethylene signaling is still largely unknown. Genome wide phospho-peptide survey in 3-day old etiolated seedlings treated with air or ethylene was done previously [11], we found the phosphorylation of CBP20 is highly regulated by ethylene gas (Fig 1). In the absence of ethylene, no phosphorylated peptides of CBP20 were detected, while in the presence of ethylene gas, 17 spectrum counts of phosphorylation peptide (239aa-253aa) was detected. Further genetics study demonstrated that the phosphorylation of CBP20 is involved in the growth of root in the presence of ethylene (Fig 1C–1E).

CBP20 is a subunit of CBC complex, which is vital for plant development. Previous study has demonstrated that CBP80, the other subunit of CBC is involved in the regulation of hypocotyl in ethylene signaling through regulation the biogenesis of small RNAs [47]. In mammalians, it has demonstrated that growth factors mTORC1 kinase regulated S6 kinases able to phosphorylates CBP80, activating the CBC affinity for 7mG [48, 49]. However, no evidence has shown CBC complex is regulated by phosphorylation in response to hormones. Here, for the first time we provide evidence showing that CBP20 Ser245 site is highly phosphorylated with ethylene treatment. The constitutive phosphorylated *CBP20*, while not the constitutive non-phosphorylated *CBP20* is able to rescue the ethylene root phenotype of *cbp20*, strongly suggesting that the phosphorylation of CBP20 is involved in ethylene response. Yet, how CBP20 phosphorylation occurs in the presence of ethylene is still undetermined. In our precious study of phosphopeptides in *etr1-1* mutant with or without ethylene treatment, no phosphorylation was detected for CBP20, showing that the phosphorylation of CBP20 is ethylene dependent. Therefore, the identification of kinases that regulate CBP20 phosphorylation specifically in the presence of ethylene will be an immediately interest.

### Ethylene regulated and CBP20 dependent miRNAs in roots

CBC complex regulate many aspects of biological processes including transcription regulation, pre-mRNA splicing, pre-mRNA 3’end processing, miRNA biogenesis, mRNA stability, mRNA and snRNA nuclear export, the pioneer round of translation and nonsense-mediated RNA decay [28]. However, no evidence has shown that CBP20 is involved in ethylene response. In our study, through high throughput sequencing for small RNAs and mRNAs in different plants treated with or without ethylene, we identified ethylene regulated miRNAs. In addition, we found that CBP20 regulates many species of miRNA expressions in response to ethylene with a tissue specific manner (Fig 2). miRNAs are involved in many different aspects of plants. Specifically in plant hormones, such as miR160 targets to several ARF family members to activate auxin signaling pathway for root cap formation [50]; miR159 targets to *MYB33* to activate ABA signaling pathway for seed germination [30]. In ethylene signaling pathway, it have been reported that EIN3 represses miR164 transcription and up regulates the transcript level of *NAC2* to regulate leaf senescence [51]. Here we provide evidence showing for the first time that miRNAs are differentially regulated by ethylene in a tissue specific manner (Fig 2A and 2B), and many of the differential regulations are abolished in *cbp20* mutant (Fig 2A and 2B). Previous studies have shown that CBP20 is required for the biogenesis of many miRNAs [30]. Interestingly, our data showed that some miRNA species are down regulated in *cbp20* mutant, which indicating non-CBP20 dependent miRNA biogenesis is potentially involved in ethylene response.

By comparing the miRNAs in Col-0 and in that of *cbp20*, we found many miRNA species are up regulated in *cbp20* in the presence of ethylene. One possibility is that the precursors of those miRNAs are elevated by ethylene, resulting in the elevation of their miRNAs. Alternatively, CBP20 independent miRNA biogenesis machinery is elevated in the presence of ethylene, resulting in the increase of the miRNAs. However, recently study has shown that small RNA biogenesis machinery component Dicers are not involved in ethylene response [15]. Therefore, further comprehensive studies will be critical to characterize the newly identified ethylene regulated, while not CBP20 dependent miRNAs and uncover the mechanistic details that how biogenesis occurs specifically in the presence of ethylene.

### Ethylene induced CBP20 phosphorylation regulates *miR319* and its target *MYB33* in roots

MicroRNAs in plant are small RNAs, which are approximately 21 nucleotides in length. Normally, they are negative regulators of gene expression through base pairing to the complementary sequence within the target mRNAs, leading to the target mRNA degradation through RISC-mediated cleavage. In comparing to the ethylene altered small RNAs with ethylene regulated genes, we found that *miR319b* was up-regulated while *MYB33* was down-regulated in Col-0 roots with ethylene treatment, and the regulation is CBP20 dependent. Small RNA northern blot shows that *miR319* is indeed up-regulated under ethylene treatment and the regulation is dependent on CBP20 phosphorylation. However, it is well known that *MYB33* is a shared target between miR159a and miR319b. The miR159 and miR319 families are similar in sequence, but they have distinct target genes: miR159 is specific for MYB transcription factors, mainly *MYB33* and *MYB65*. In contrast, miR319 mainly targets TCP transcription factors, predominantly TCP2 and TCP4. MiR319 also targets *MYB33* and *MYB65*, but due to its low abundance, this regulation is negligible. However, in our study we provided multiple lines of evidence showing that in the ethylene response, miR319b targets *MYB33* for degradation specifically in roots, leading to the ethylene regulated *cbp20* root phenotype: (1), miR319 was specifically up regulated by ethylene in Col-0 roots, while not in *cbp20* mutant (Fig 4A). However, miR159 was not regulated by ethylene (Fig 5A); (2) *MYB33* was down regulated in Col-0 roots in response to ethylene, while not in *cbp20* roots (Fig 4C). (3) The pri-miR319b was down regulated in Col-0 roots, while not in *cbp20* roots (Fig 4B); (4) The pri-miR159a was not regulated by ethylene in Col-0 (Fig 5B); (5) In the over expression *miR319b* plants, *MYB33* was largely down regulated, while miR159 and pri-miR159a were not altered (Figs 4C, 5B and 5C); (6) Overexpression of the *mMYB33* containing mutated miR319b target site is able to recover phenotype caused by *miR319bOE* (Fig 6); (7) Our data (Fig 7) and published data has shown that miR319b targets *MYB33* for cleavage [32]. In summary, our study discovered that ethylene regulates the phosphorylation of CBP20, and the phosphorylation is required for the elevation of *miR319*, which leading to the down regulation of *MYB33* expression in roots, resulting in root growth inhibition in the presence of ethylene.

## Materials and Methods

### Plant materials and growth conditions

All mutants were in the Columbia-0 (Col-0) background, *cbp80* (CS878659), *myb33-1* (SALK_065473), *myb33-2* (SALKseq_056201) are ordered from ABRC. *cbp20* has been described in [52]. Seeds were sterilized with 4% bleach and then washed as least three times with sterilized water, then the seeds were sown on MS medium. Plants were grown in long days (16h light/8h dark) at 22°C on soil.

### Plasmid and transgenic plants construction

To construct CBP20 overexpression vectors for complementing *cbp20* mutant phenotype, the *CBP20* full length and phosphorylation site mutated CDS sequences of *CBP20* were amplified using the Phusion High-Fidelity DNA Polymerase (NEB). The PCR products were cut with KpnI and SalI, and then the corresponding fragments were ligated into the KpnI-SalI site of the pCHF3 vector to give rise to *35S*:*CBP20-YFP*, *35S*:*CBP20*^*S245A*^-*YFP*, *35S*:*CBP20*^*S245E*^*-YFP* and *35S*:*CBP20*^*S245D*^*-YFP*.

To construct vectors for yeast two-hybrid, the CDS of CBP80 was amplified using the Phusion High-Fidelity DNA Polymerase. The PCR product was cut with SalI and XbaI, and then the corresponding fragment was ligated into the SalI-SpeI site of the pDBLeu vector (Invitrogen) to give rise to pBD-*CBP80*. The CDSs of *CBP20*, *CBP20*^*S245A*^, *CBP20*^*S245E*^ and *CBP20*^*S245D*^ were amplified using the High-Fidelity DNA Polymerase. The PCR products were digested by SalI and SpeI, and the corresponding fragments were ligated into the SalI-SpeI sites of the pEXP-AD502 vector (Invitrogen) to give rise to pAD*-CBP20*, pAD*-CBP20*^*S245A*^, pAD*-CBP20*^*S245E*^ and pAD*-CBP20*^*S245D*^.

To construct miR319b overexpression vector, a 1kb genomic DNA contain the full length of pri-miR319b was amplified using the Phusion High-Fidelity DNA Polymerase. The PCR product was digested with KpnI and SalI, and then the corresponding fragment was ligated into the KpnI-SalI site of the pCHF3 vector to give rise to pCHF3-miR319b. All the sequences above were verified by sequencing.

The binary constructs were introduced into *Agrobacterium tumefaciens* strain GV3101 by electroporation and then introduced into Col-0 or *cbp20* mutant plants by the floral dip method [53]. Transgenic plants were screened on MS plates in the presence of 50 mg/L kanamycin, and homozygous lines were verified by antibiotic selection. For each construct, multiple independent lines were examined with similar results, and as least one representative line was shown.

### Phosphopeptide identification

The data has been collected from previous study and the calculation was also followed the method as published [11].

### Triple response phenotype measurement

*Arabidopsis* seeds were sown on MS medium plates with or without addition of 1 μM or 10 μM 1-aminocyclopropane-1-carboxylic acid (ACC, Sigma), the biosynthetic precursor of ethylene. After 3 days of cold treatment, the plates were wrapped in foil and kept in 22°C dark chamber for 3 days. The hypocotyls and roots were measured using NIH Image (http://rsb.info.nih.gov/nih-image/).

### Yeast two-hybrid assay

The yeast two-hybrid assay was performed according to the ProQuest^™^ Two-Hybrid System (Invitrogen). Briefly, pBD-*CBP80* and pAD-*CBP20*, -*CBP20*^*S245A*^, *-CBP20*^*S245E*^ or *-CBP20*^*S245D*^ were co-transformed into the yeast strain Mav203 (Invitrogen). The transformants were grown on SD/-Trp-Leu medium or SD/-Trp-Leu-His with 10mM 3AT dropout medium. The transformants growing on SD/-Trp-Leu-His with 10mM 3AT dropout medium indicates interaction between corresponding proteins. Primers used in this assay were listed in S5 Table.

### Subcellular localization

The seedlings of *35S*:*CBP20-YFP*, *35S*:*CBP20*^*S245A*^-*YFP*, *35S*:*CBP20*^*S245E*^*-YFP* and *35S*:*CBP20*^*S245D*^*-YFP* transgenic plants were grown on MS medium with or without addition of 10 μM ACC in dark for 3 days in 22°C. Then the YFP fluorescence of root tips was observed under Zeiss LSM 710 Confocal microscopy.

### RNA extraction and real-time PCR

*Arabidopsis* seeds were grown on MS medium in the air-tight containers in the dark at 22°C supplied with a flow of hydrocarbon-free air (Zero grade air, AirGas) for 3 days. The plants tissues were harvest after with continually flow of hydrocarbon-free air or hydrocarbon-free air with 10 parts per million (ppm) ethylene gas for 4 hours as previously described [7].

Total RNA was extracted using a RNeasy Plant Kit (Qiagen) from 3 days etiolated seedlings treated with air or 4 hours ethylene gas. First-strand cDNA was synthesized using Superscript III First-Strand cDNA Synthesis Kit (Invitrogen). Real time PCR was performed with the LightCycler 480 SYBR Green I Master (Roche) following the manufacturer’s instructions. PCR reactions were performed in triplicate on a Roche 96 Thermal cycler. The expression level was normalized to *UBQ10* control.

### mRNA and small RNA library construction

Total RNA was isolated from roots or shoots of 3-day old etiolated seedlings treated with air or 4 hours ethylene gas using TRIzol reagent (Invitrogen). For mRNA library construction, in briefly, the mRNA was isolated by NEBNext Poly(A) mRNA Magnetic Isolation Module and fragmented at 94°C for 15mins. Then the cDNA was synthesis by NEBNext Ultra Directional RNA Library Prep Kit for Illumina. The PCR reactions were conducted by using different index primers (NEBNext Multiplex Oligos for Illumina). The PCR products were purified by Agencourt AMPure XP Beads (Beckman Coulter). The quality of the libraries was assessed by Bioanalyzer (Agilent High Sensitivity Chip). The libraries then were sequenced on Hiseq 4000 Systems (Illumina).

For small RNA library construction, in briefly, the cDNAs were synthesized using NEBNext Small RNA Library Prep Set for Illumina. The PCR reaction was amplified by different Index primers and the PCR products were first purified by the Agencourt AMPure XP Beads, and then selected the size using 6% PolyAcrylamide Gel. The ~140 bp bands corresponding to miRNAs were isolated. The library quality was assessed on Bioanalyzer (Agilent High Sensitivity Chip). The libraries were sequenced on Hiseq 4000 Systems (Illumina) after assessed on Bioanalyzer.

### miRNA identification

miRNA prediction pipeline was written by Python scripting language. High-quality small RNA reads were obtained from raw reads through filtering out poor quality reads and removing adaptor sequences using FASTX toolkit [54]. Adaptor-trimmed unique sequences were aligned to TAIR10 *Arabidopsis* genome using bowtie [55] and structural RNAs such as tRNA, rRNA, snRNA, and snoRNA were excluded. The perfect matched reads between 18–28 nucleotides (nts) in length were selected. To obtain the precursor sequences, potential miRNA sequences (reads ≥ 50) were extended upstream and downstream of 100 to 500 nts with a step size of 100 nts. Each putative precursor sequence was folded using RNA fold from Vienna RNA software package [56], and the potential miRNA* sequences were selected with mismatch ratio of 0.3 or less. The region of these putative precursor sequences with addition of 15 nts marginal sequences were re-folded using RNA fold to check whether miRNA/miRNA* duplex was suitable for primary criteria for annotation of plant miRNAs [57]. The miRNA candidates were essentially grouped into families by mature sequence similarity and/or loci. Using the miRNA annotation information of *Arabidopsis thaliana* in miRBase 21 (http://www.mirbase.org), all members of miRNA candidate families of the known miRNAs were selected.

### miRNA target prediction

The putative target sites of miRNAs were identified by aligning mature miRNA sequences with the *Arabidopsis* cDNA sequences using TargetFinder (http://carringtonlab.org/resources/targetfinder). miRNA targets were computationally predicted essentially as described [58–60]. Briefly, potential targets from FASTA searches were scored using a position-dependent, mispairing penalty system. Penalties were assessed for mismatches, bulges, and gaps (+1 per position) and G:U pairs (+0.5 per position). Penalties were doubled if the mismatch, bulge, gap, or G:U pair occurred at positions 2 to 13 relative to the 5’-end of the miRNAs. Only one single-nucleotide bulge or single-nucleotide gap was allowed, and the targets with penalty scores of six or less were considered to be putative miRNA targets.

### RNA-seq data analysis

RNA-seq raw reads were aligned to TAIR10 genome release using Top Hat version 2.0.9 [61] with default parameters. Differential expressed genes were calculated by Cufflinks version 2.2.1 following the workflow with default parameters [62]. Differentially expressed genes were those for which relative fold change values of larger than 1.5 and RPKM value larger than 1 were observed. To evaluate reproducibility of the RNA-seq data, the expression levels between two replicates for each sample and conditions were compared for all genes with FPKM > 0.5 in both replicates. The log2 transformed FPKM values (log2 (FPKM + 1) was calculated, then R scripts were used to analyze the correlation between biological replicates.

### Small RNA northern blot

Total RNA was isolated from root of 3 days etiolated seedlings treated with air or 4 hours ethylene gas using TRIzol reagent (Invitrogen). 10ug RNA of each sample was separated on 15% denaturing 8M urea-PAGE gel and then transferred and UV crosslinked onto BrightStar^®^-Plus Positively Charged Nylon Membrane (Ambion). The membrane was pre-hybridized by ULTRAhyb^®^-Oligo Hybridization Buffer. miRNA probes were end-labelled by T4 Polynucleotide Kinase (NEB) with r-P^32^ ATP. The membrane was hybridized with probe overnight and then wash by 2xSSC for two times. Then the membrane was exposed to a phosphor imager screen and the relative abundance levels were measured by ImageQuant TL software.

### Modified 5’RLM-RACE of cleaved miRNA targets

5’RLM-RACE was performed following the manufacturer’s instructions of FirstChoice RLM-RACE Kit (Ambion). Briefly, total RNA (10 μg) from root of Col-0 and *miR319b OE* line was directly ligated to the 5’RACE Adapter by T4 RNA ligase (Ambion). cDNA was synthesized using Superscript III First-Strand cDNA Synthesis Kit (Invitrogen) use Oligo (dT) primer. Gene-specific reaction was first done with the 5’RACE Outer Primer and gene-specific primer *MYB33* SpeI-R. Then the PCR product was purified and performed by the second round of PCR using 5’RACE inner Primer and gene-specific primer *MYB33* SpeI-R (S5 Table). The 5RLM-RACE product was gel purified, digested with Sal I and Spe I and then cloned into pDBLeu vector for sequencing.

### *N*. *benthamiana* transient expression assay

Transient expression assay in *N*. *benthamiana* were performed by infiltrating 4-week-old *N*. *benthamiana* plants with *Agrobacterium* containing *MYB33* or mutated *MYB33* CDS with or without *Agrobacterium* harbouring constructs containing the *miR319b* precursor. Leaf tissue was collected 3 days later for RNA and protein analysis. For luciferase assay, *Agrobacterium* containing *MYB33* or mutated m*MYB33* CDS fused to 35S::LUC 3’UTR with or without *Agrobacterium* harbouring constructs containing the *miR319b* precursor were injected in to *N*. *benthamiana* plants. After 3 days, The leaves were sprayed with 500 μM luciferin (Promega, Madison, Wisconsin) and placed in the dark for 5 min. Luciferase activity was observed using NightOWL LB 983 *in vivo* Imaging System (Berthold, Oak Ridge, Tennessee). Primers used in this study were listed in S5 Table.

## Supporting Information

S1 Fig S245 of CBP20 is phosphorylated and CBP20 is conserved in different species. (A) The phosphopeptide detected by Mass Spec in 3-day old etiolated seedlings treated with ethylene. (B) Protein alignment of CBP20 from various species. The alignment was generated using DNAMAN with default parameters. The positions of conserved residues are shown in black, and similar residues are shown in turquoise and magenta, respectively. The red star indicates the Ser245 site. The following sequences were used to establish the alignment: *Arabidopsis thaliana* (At), BAB10987; *Nicotiana tabacum* (Nt), ACY02034; *Glycine max* (Gm), XP_006594834; *Solanum tuberosum* (St), ACY07775; *Populus trichocarpa* (Pt), XP_006372745; *Vitis vinifera* (Vv), XP_010657003; *Oryza sativa* (Os), AAP33448; *Zea mays* (Zm), ACG37816; *Hordeum vulgare* (Hv), ACL83596; *Sorghum bicolor* (Sb), XP_002454163.
(TIF)

S2 Fig (A) Schematic diagrams show the construction of CBP20 phosphorylation site mutation vectors. (B) Triple response phenotype of *cbp80* mutant in roots on 10μM ACC plate for 3 days. The Col-0 and *cbp80* mutant were grown on MS medium with or without 10μM ACC for 3 days before measurement. (C) The interaction between CBP80 and CBP20, constitutive dephosphorylated of CBP20 (CBP20^S245A^) or constitutive phosphorylated of CBP20 (CBP20^S245E^ and CBP20^S245D^) by yeast two-hybrid assay. (D) The cell localization of CBP20, CBP20^S245A^ and constitutive CBP20^S245E^ and CBP20^S245D^ proteins fused with YFP. The photos were taken by confocal laser scanning microscope in root tips of 3-day old etiolated seedlings treated with air or ethylene.
(TIF)

S3 Fig Length distribution of genome-matching reads representing small RNAs with indicated 5'-nucleotide in roots (A) and in shoots (B). Reads matching rRNA, tRNA, and snRNA are excluded. Labels of each experiment were made by combination of Colombia (Col), *cbp20* mutant line (*cbp20*), root (RT), shoot (ST), air (a), and ethylene-treated (C). Replicates are indicated by numbers at the end. X-axis: Length with 5'-nucleotide identity, Y-axis: Labels of each experiment, Z-axis: Genome-matching reads (CP10M normalized), CP10M: count per 10 million mapped reads.
(TIF)

S4 Fig RNA-seq quality detection of differential regulated genes in Col-0 and *cbp20*. (A) Scatter plots of gene expression level show quality of RNA-seq data of shoots and roots in Col-0 treated with air (left panel) and ethylene gas (right panel). (B) Scatter plots of gene expression level show quality of RNA-seq data of shoots and roots in *cbp20* mutant treated with air (left panel) and ethylene gas (right panel).
(TIF)

S5 Fig Gene ontology analysis of genes altered by ethylene specifically in roots, shoots or shared by roots and shoots. (A) GO terms analysis of specific differential regulated genes in col root after ethylene treatment. (B) GO terms analysis of specific differential regulated genes in col shoot after ethylene treatment. (C) GO terms analysis of overlap differential regulated genes in col root and shoot after ethylene treatment.
(TIF)

S6 Fig miR319b is involved in root growth in the presence of ethylene. (A-B) The relative hypocotyl length (A) and root length (B) (ACC/MS) of 3-day old etiolated seedlings of *miR319b OE*, *myb33* mutants and Col-0 plants grown on MS or MS with or without 1μm ACC. Different letters were used to indicate statistically significance difference (P≤0.05). (C) Cleavage sites and cleavage event ratios of *MYB33* mRNA in Col-0 and *miR319b OE* lines by RLM-RACE. Arrows indicate positions and proportions of clones mapped to the cleavage sites. “a” indicates no statistic significant difference. “b” indicates statistic significant difference.
(TIF)

S7 Fig *MYB33* is the target of miR319b. (A) Diagram showing the mutations were introduced into *MYB33*, and the mutation sites localized to the target site of miR319b. (B) qRT-PCR to examine the expression of pri-miR319b in *miR319bOE* plants and *mMYB33OE/miR319bOE* plants, showing that *MYB33* is target of miR319b. The RNAs used in qRT-PCR were extracted from the roots of 3-old etiolated seedlings from the plants indicated in the figure. (C) qRT-PCR to examine the expression of *TCPs*, the known target of miR319, showing that the expression of *TCPs* is not affected by ethylene in Col-0. (D) qRT-PCR to examine the expression of *TCP24* in *miR319bOE and mMYB33OE/miR319bOE* showing that the expression of *TCP24* is not affected by *mMYB33*. (E) qRT-PCR to examine the expression of other TCPs, showing they have the similar expression pattern as TCP24 in *miR319bOE*.
(TIF)

S8 Fig *MYB33* is a target of miR319b. (A) *MYB33* and *mMYB33* were detected by qRT–PCR. *UBQ10* was used as a loading control. (B) Protein levels were detected by western blot with HA antibody. Ponceau S staining was used as a loading control. Agrobacterium-mediated transient co-expression assayin tobacco leaves using YFP-HA-tagged *MYB33* or mutated *MYB33* (*mMYB33*) CDS with or without miR319b. The infected leaves were harvest for qRT-PCR and western blot assay. Proteins of MYB33 and mMYB33 were detected by anti-HA antibody.
(TIF)

S1 Table Differential expression of miRNAs regulated by ethylene in Col-0 roots. (XLSX)

S2 Table Differential expression of miRNAs regulated by ethylene in *cbp20* roots. (XLSX)

S3 Table Differential expression of miRNAs regulated by ethylene in Col-0 shoots. (XLSX)

S4 Table Differential expression of miRNAs regulated by ethylene in *cbp20* shoots. (XLSX)

S5 Table Primers used in this study. (XLSX)

S1 Dataset Differential regulated genes in Col-0 root by ethylene treatment. (XLSX)

S2 Dataset Differential regulated genes in *cbp20* root by ethylene treatment. (XLSX)

S3 Dataset Differential regulated genes in Col-0 shoot by ethylene treatment. (XLSX)

S4 Dataset Differential regulated genes in *cbp20* shoot by ethylene treatment. (XLSX)

S5 Dataset CBP20 dependent, ethylene up-regulated genes in Col-0 root. (XLSX)

S6 Dataset CBP20 dependent, ethylene down-regulated genes in Col-0 root. (XLSX)

S7 Dataset The targets of ethylene up-regulated miRNAs in Col-0 root (t score less than 5). (XLSX)

S8 Dataset The targets of ethylene down-regulated miRNAs in Col-0 root (t score less than 5). (XLSX)
