# Supplementary figures and images for: Phosphorylation of CBP20 Links MicroRNA to Root Growth in the Ethylene Response
Source: PLoS Genet. 2016 Nov 21;12(11):e1006437. doi: 10.1371/journal.pgen.1006437 (PMC5147770; doi:10.1371/journal.pgen.1006437)

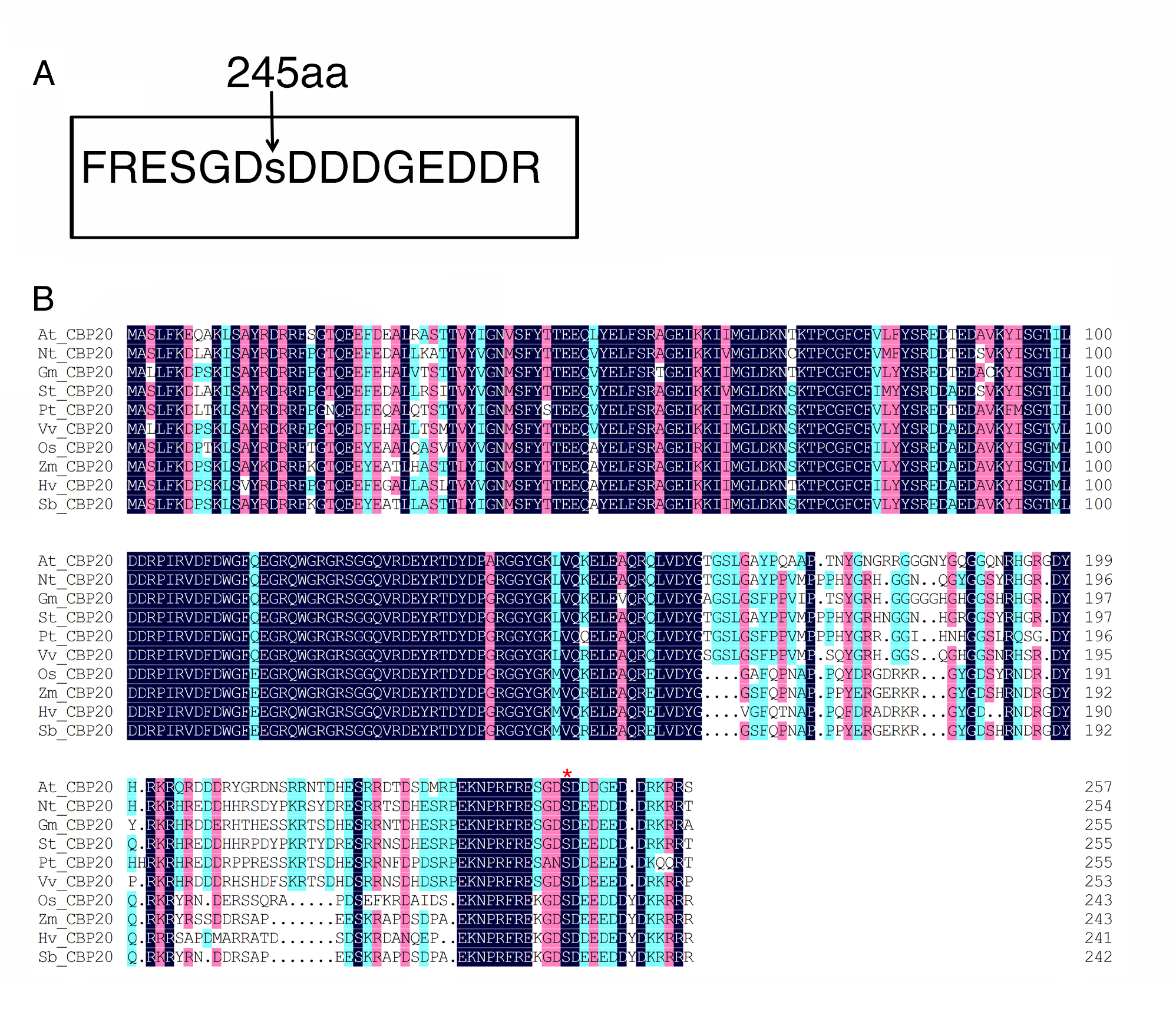

Supplement: S1 Fig — (A) The phosphopeptide detected by Mass Spec in 3-day old etiolated seedlings treated with ethylene. (B) Protein alignment of CBP20 from various species. The alignment was generated using DNAMAN with default parameters. The positions of conserved residues are shown in black, and similar residues are shown in turquoise and magenta, respectively. The red star indicates the Ser245 site. The following sequences were used to establish the alignment: Arabidopsis thaliana (At), BAB10987; Nicotiana tabacum (Nt), ACY02034; Glycine max (Gm), XP_006594834; Solanum tuberosum (St), ACY07775; Populus trichocarpa (Pt), XP_006372745; Vitis vinifera (Vv), XP_010657003; Oryza sativa (Os), AAP33448; Zea mays (Zm), ACG37816; Hordeum vulgare (Hv), ACL83596; Sorghum bicolor (Sb), XP_002454163. (TIF) [file pgen.1006437.s001.tif]

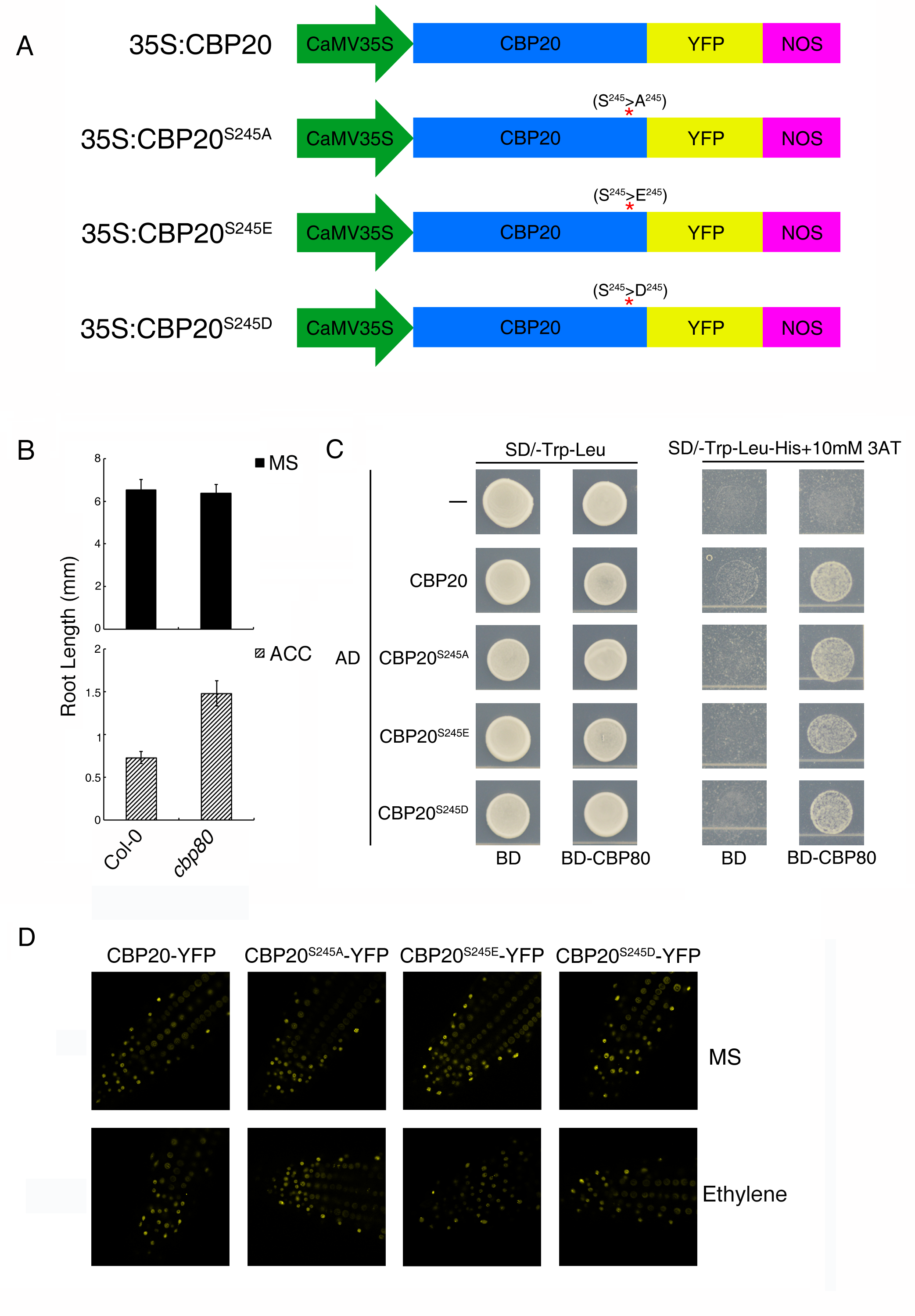

Supplement: S2 Fig — (A) Schematic diagrams show the construction of CBP20 phosphorylation site mutation vectors. (B) Triple response phenotype of cbp80 mutant in roots on 10μM ACC plate for 3 days. The Col-0 and cbp80 mutant were grown on MS medium with or without 10μM ACC for 3 days before measurement. (C) The interaction between CBP80 and CBP20, constitutive dephosphorylated of CBP20 (CBP20S245A) or constitutive phosphorylated of CBP20 (CBP20S245E and CBP20S245D) by yeast two-hybrid assay. (D) The cell localization of CBP20, CBP20S245A and constitutive CBP20S245E and CBP20S245D proteins fused with YFP. The photos were taken by confocal laser scanning microscope in root tips of 3-day old etiolated seedlings treated with air or ethylene. (TIF) [file pgen.1006437.s002.tif]

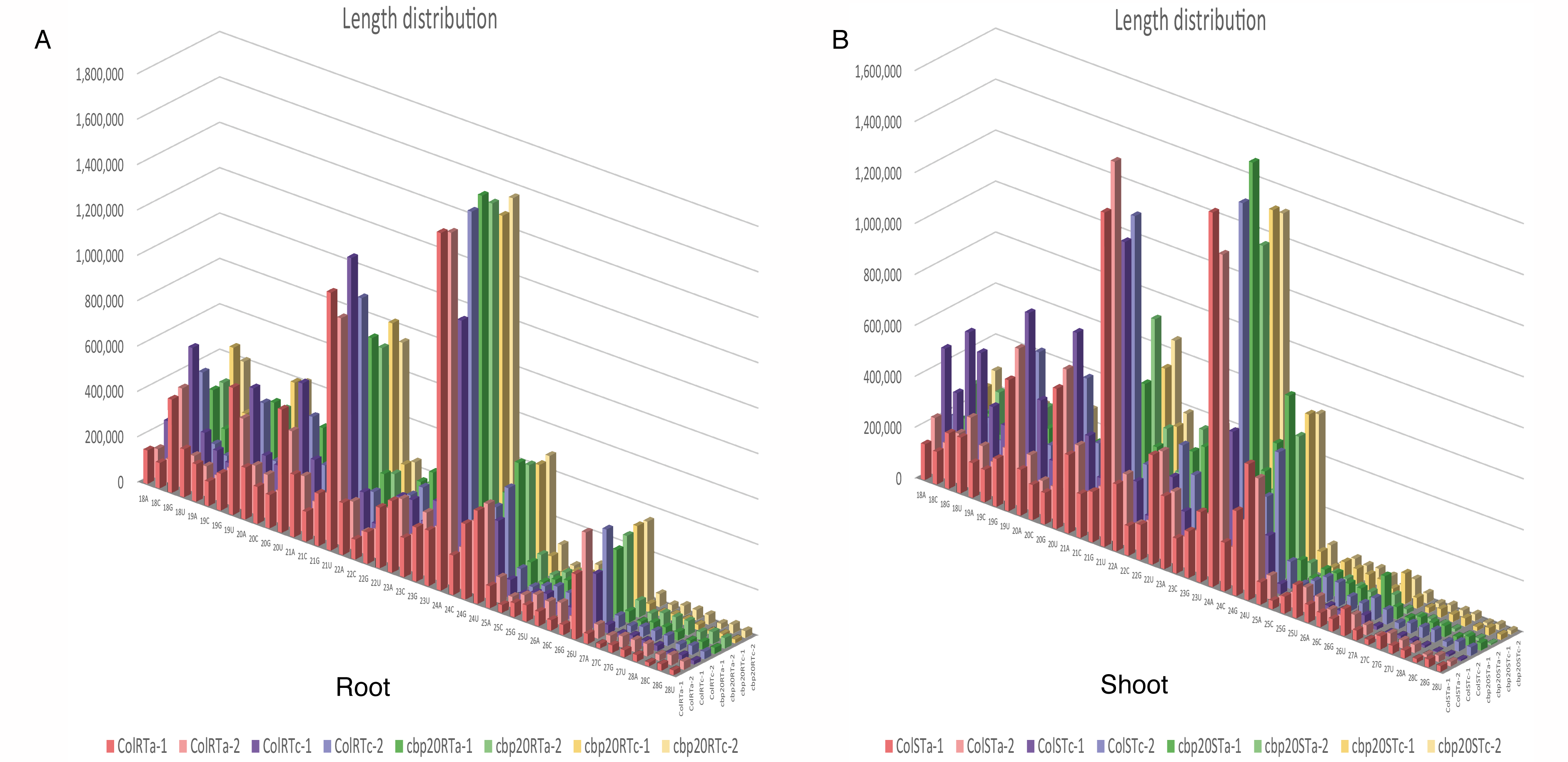

Supplement: S3 Fig — Length distribution of genome-matching reads representing small RNAs with indicated 5'-nucleotide in roots (A) and in shoots (B). Reads matching rRNA, tRNA, and snRNA are excluded. Labels of each experiment were made by combination of Colombia (Col), cbp20 mutant line (cbp20), root (RT), shoot (ST), air (a), and ethylene-treated (C). Replicates are indicated by numbers at the end. X-axis: Length with 5'-nucleotide identity, Y-axis: Labels of each experiment, Z-axis: Genome-matching reads (CP10M normalized), CP10M: count per 10 million mapped reads. (TIF) [file pgen.1006437.s003.tif]

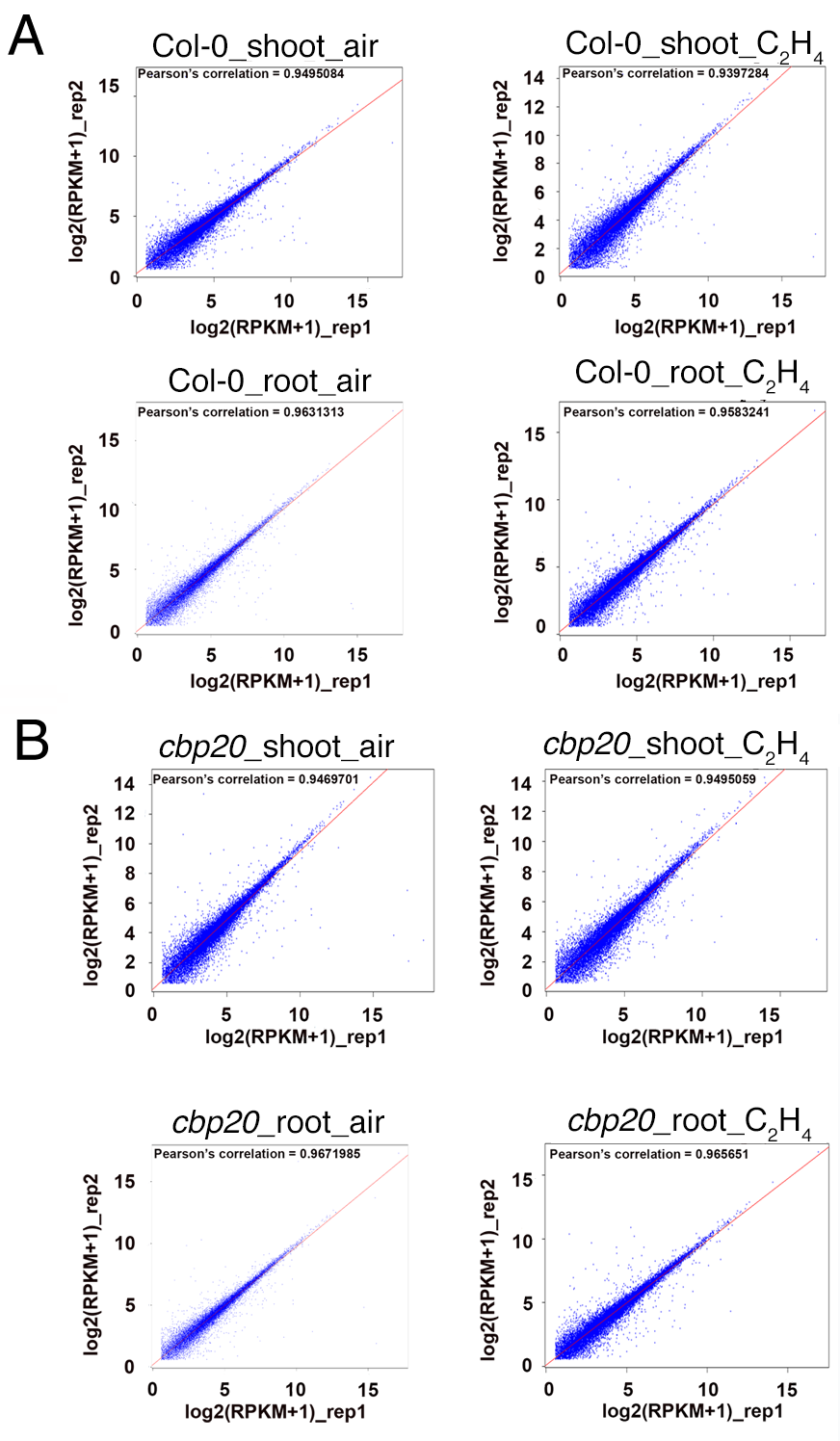

Supplement: S4 Fig — (A) Scatter plots of gene expression level show quality of RNA-seq data of shoots and roots in Col-0 treated with air (left panel) and ethylene gas (right panel). (B) Scatter plots of gene expression level show quality of RNA-seq data of shoots and roots in cbp20 mutant treated with air (left panel) and ethylene gas (right panel). (TIF) [file pgen.1006437.s004.tif]

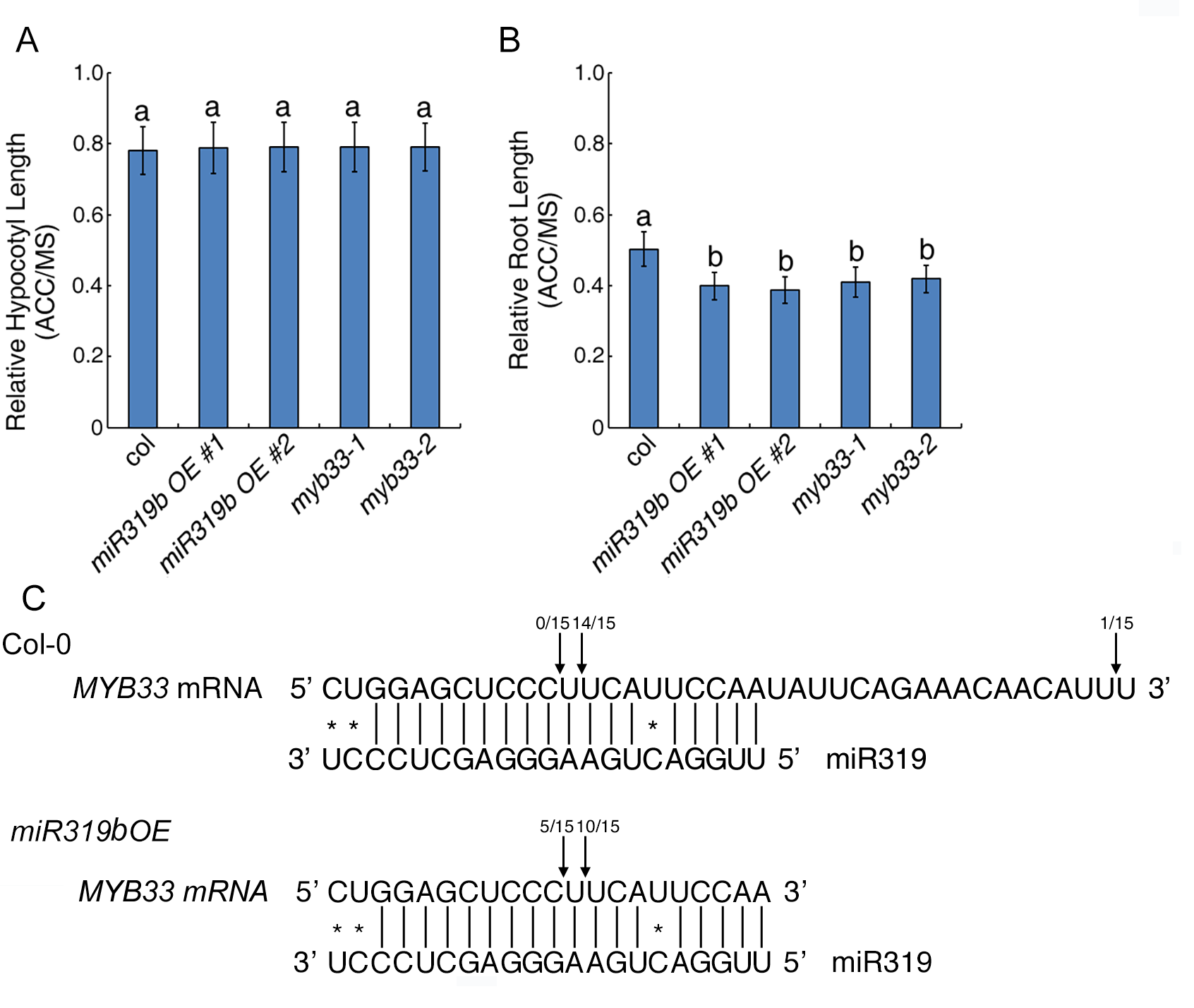

Supplement: S6 Fig — (A-B) The relative hypocotyl length (A) and root length (B) (ACC/MS) of 3-day old etiolated seedlings of miR319b OE, myb33 mutants and Col-0 plants grown on MS or MS with or without 1μm ACC. Different letters were used to indicate statistically significance difference (P≤0.05). (C) Cleavage sites and cleavage event ratios of MYB33 mRNA in Col-0 and miR319b OE lines by RLM-RACE. Arrows indicate positions and proportions of clones mapped to the cleavage sites. “a” indicates no statistic significant difference. “b” indicates statistic significant difference. (TIF) [file pgen.1006437.s006.tif]

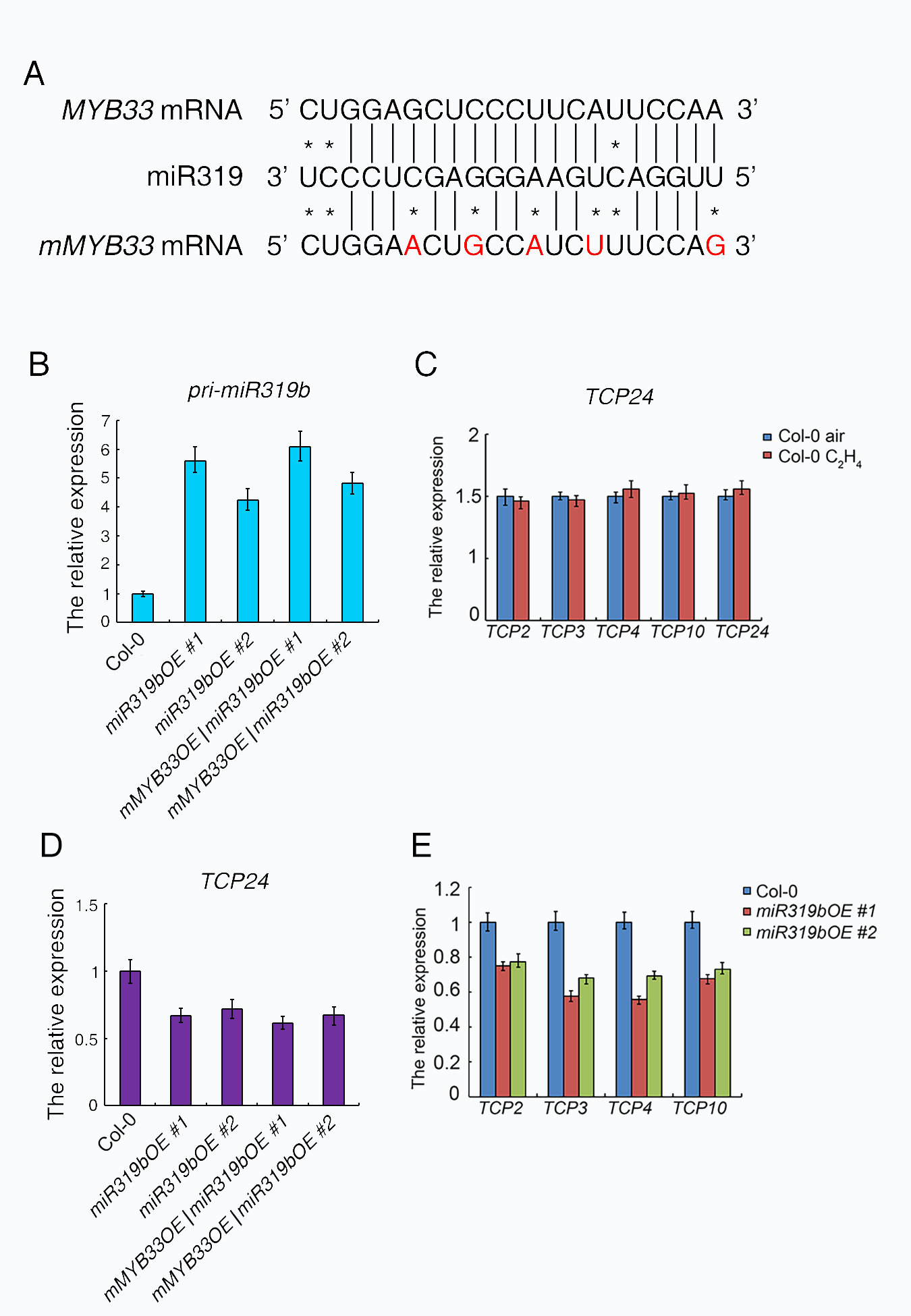

Supplement: S7 Fig — (A) Diagram showing the mutations were introduced into MYB33, and the mutation sites localized to the target site of miR319b. (B) qRT-PCR to examine the expression of pri-miR319b in miR319bOE plants and mMYB33OE/miR319bOE plants, showing that MYB33 is target of miR319b. The RNAs used in qRT-PCR were extracted from the roots of 3-old etiolated seedlings from the plants indicated in the figure. (C) qRT-PCR to examine the expression of TCPs, the known target of miR319, showing that the expression of TCPs is not affected by ethylene in Col-0. (D) qRT-PCR to examine the expression of TCP24 in miR319bOE and mMYB33OE/miR319bOE showing that the expression of TCP24 is not affected by mMYB33. (E) qRT-PCR to examine the expression of other TCPs, showing they have the similar expression pattern as TCP24 in miR319bOE. (TIF) [file pgen.1006437.s007.tif]

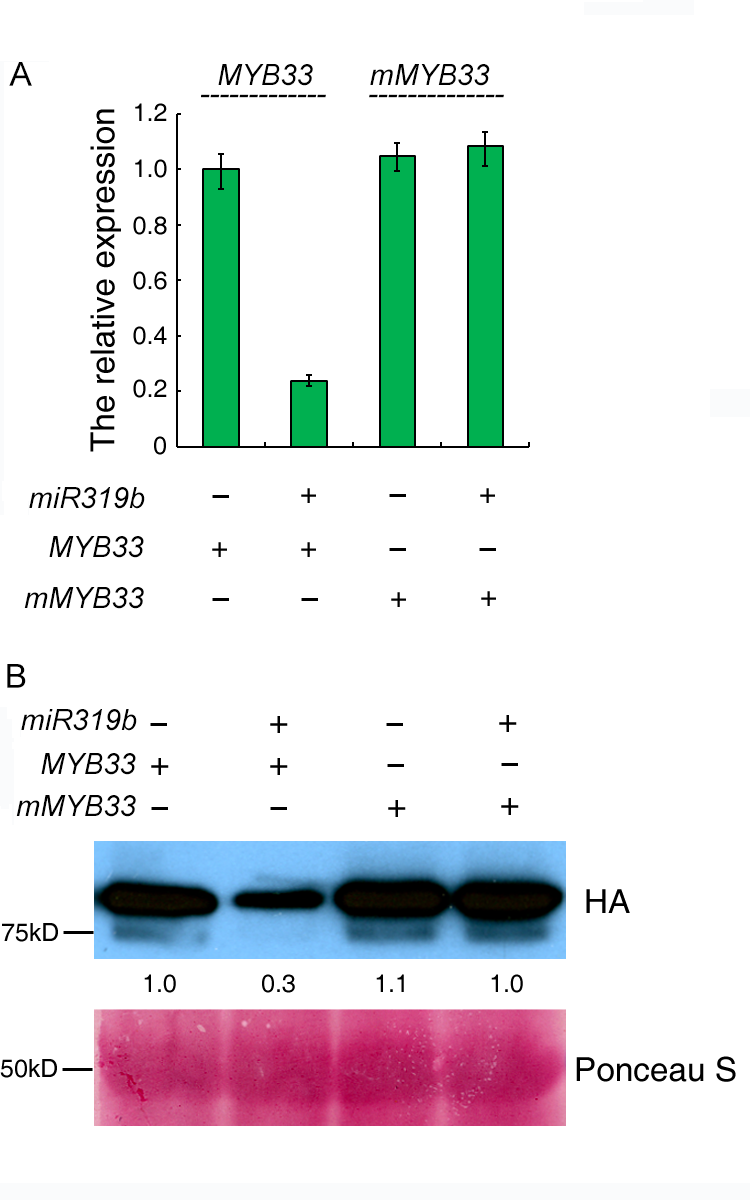

Supplement: S8 Fig — (A) MYB33 and mMYB33 were detected by qRT–PCR. UBQ10 was used as a loading control. (B) Protein levels were detected by western blot with HA antibody. Ponceau S staining was used as a loading control. Agrobacterium-mediated transient co-expression assayin tobacco leaves using YFP-HA-tagged MYB33 or mutated MYB33 (mMYB33) CDS with or without miR319b. The infected leaves were harvest for qRT-PCR and western blot assay. Proteins of MYB33 and mMYB33 were detected by anti-HA antibody. (TIF) [file pgen.1006437.s008.tif]
